# Efficacy and safety assessment of acupuncture and nimodipine to treat mild cognitive impairment after cerebral infarction: a randomized controlled trial

**DOI:** 10.1186/s12906-016-1337-0

**Published:** 2016-09-13

**Authors:** Shuhua Wang, Hongling Yang, Jie Zhang, Bin Zhang, Tao Liu, Lu Gan, Jiangang Zheng

**Affiliations:** 1 Department of Moxibustion and Massage, Tianjin University of Traditional Chinese Medicine, Tianjin, 300193 China; 2 Department of Acupuncture, The First Teaching Hospital of Tianjin University of Traditional Chinese Medicine, Tianjin, 300193 China

**Keywords:** Acupuncture, Cerebral infarction, Mild cognitive impairment, Montreal Cognitive Assessment scale, Nimodipine

## Abstract

**Background:**

Cerebral infarction frequently leads to mild cognitive impairment (MCI). Prompt management of MCI can prevent vascular dementia and improve patient outcome. This single center randomized controlled trial aims to investigate the efficacy and safety of acupuncture and nimodipine to treat post-cerebral infarction MCI.

**Methods:**

A total of 126 Chinese patients with post-cerebral infarction MCI recruited from the First Teaching Hospital of Tianjin University of Traditional Chinese Medicine between April 2013 and June 2014 were randomized at 1:1: 1 ratio into nimodipine alone (30 mg/time and 3 times daily), acupuncture alone (30 min/time, 6 times/week), and nimodipine + acupuncture groups. The treatments were 3 months. Cognitive function was evaluated using Montreal Cognitive Assessment (MoCA) scale at enrollment interview, at the end of 3-month therapy, and at the post-treatment 3-month follow-up.

**Results:**

The per-protocol set included 39, 40, and 40 patients from nimodipine alone, acupuncture alone, and the combination group, respectively, was analyzed. Intra-group comparison revealed that MoCA score at the follow-up improved significantly by 15.8 ± 10.9, 20.9 ± 13.8 %, and 30.2 ± 19.7 % compared with the baseline MoCA for nimodipine alone, acupuncture alone, and the combination group, respectively. Inter-group comparison demonstrated that the combination therapy improved MoCA score (5.5 ± 2.2) at significantly higher extent than nimodipine alone (3.1 ± 1.8) and acupuncture alone (4.3 ± 2.3) at the follow-up (All *P* < 0.05), and significantly higher proportion of patients in acupuncture alone group (80 %) and the combination therapy group (90 %) than in nimodipine alone group (56.4 %) showed ≥12 % MoCA score improvement compared with the baseline MoCA (All *P* < 0.05). No adverse event was reported during the study.

**Conclusion:**

Acupuncture may be used as an additional therapy to conventional pharmacological treatment to further improve the clinical outcomes of patients with post-cerebral infarction MCI.

**Trial registration:**

The study was registered at the Chinese Clinical Trial Registry (http://www.chictr.org.cn/, Unique Identifier: ChiCTR-IOR-15007366). The date of registration is November 4, 2015.

**Electronic supplementary material:**

The online version of this article (doi:10.1186/s12906-016-1337-0) contains supplementary material, which is available to authorized users.

## Background

Cerebrovascular disease such as stroke often causes cognitive dysfunction ranging from mild cognitive impairment (MCI) to dementia. The prevalence of post-stroke cognitive impairment has been reported to be 20–80 % in patients with different nationality and races according to different diagnostic criteria [1]. Approximately 23–30 % of patients with stroke will develop MCI or dementia within 4 years after the stroke [2].

Management of post-stroke cognitive impairment has not been standardized. Previous clinical trials have shown that drugs that are usually used to treat Alzheimer’s disease, such as the cholinesterase inhibitors donepezil, galantamine, and rivastigmine, and the noncompetitive N-methyl-D-aspartate receptor antagonist memantine, were associated with neurological benefits in patients with post-stroke cognitive impairment [3–9]. However, because of uncertain effect of the drugs on global and daily function, none of the drugs has achieved regulatory approval for the treatment of vascular cognitive impairment (VCI) [10].

Nimodipine, which is a 1,4-dihydropyridine-derivative calcium channel blocker, can reduce the severity of arterial spasm-induced ischemic neurologic deficits when it is administered within 96 h of the onset of subarachnoid hemorrhage [11]. Recent clinical studies further support its beneficial effects on neurological outcomes in patients with subarachnoid hemorrhage [12]. In a systematic review of double-blinded randomized trials, nimodipine was found to have short-term benefit in terms of clinical global impression and cognitive function but not of activities of daily living in patients with VCI [13]. Although the efficacy of nimodipine in dementia, such as Alzheimer’s disease and VCI, is still uncertain, it is currently commonly used for VCI and dementia in China and several continental European countries.

In addition to the pharmacological therapies, acupuncture has also been explored to treat VCI [14]. A recent systematic meta-analysis including 691 participants pooled from 12 randomized controlled trials has shown that combination therapy of acupuncture plus other conventional therapy improved mini-mental state examination (MMSE) score significantly in patients with vascular MCI [14]. However, all the included clinical trials appear to have low quality in methodology and high risk of bias [14]. Here, this study aims to conduct a randomized trial including 126 patients with post-stroke MCI to compare the efficacy of combination therapy of acupuncture plus nimodipine versus acupuncture alone and nimodipine alone. Montreal Cognitive Assessment (MoCA) scoring system was used to evaluate the patient outcomes.

## Methods

### Trial design

This single center randomized controlled trial with 1:1:1 allocation was conducted in the First Teaching Hospital of Tianjin University of Traditional Chinese Medicine from April 2013 to June 2014. This trial was conducted in accordance with the principles of the Declaration of Helsinki. The study protocol has been approved by the Institutional Review Board of Tianjin University of Traditional Chinese Medicine (No. TYLL2013 [K] 002). The study was registered at the Chinese Clinical Trial Registry (http://www.chictr.org/cn, Unique Identifier: ChiCTR-IOR-15007366). Written informed consent was obtained from all patients.

### Patients

Patients that met all of the following inclusion criteria were enrolled: 1) aged 50 to 80 years; 2) a confirmed history of cerebrovascular ischemia based on the results from cerebral perfusion computed tomography or magnetic resonance imaging; 3) a recent episode of cerebral infarction that occurred 0.5 to 6 months prior to the enrollment interview; 4) a Modified Ashworth Scale score of muscle tone ≥ 3; 5) no severe language and cognitive disorder but presenting MCI with a MoCA score < 26; [15] the definition of MCI in this study was that the cognitive impairment was not severe enough to interfere with everyday life; [16] 6) was literate and able to read and understand the informed consent form; 7) voluntarily signed the informed consent form. Patients that met any one of the following exclusion criteria were excluded: 1) transient ischemic attack; 2) presenting cerebral hemorrhage or other disease-associated cognitive impairment; 3) severe hearing, vision, and/or cognitive dysfunction; 4) a history of mental disease; 5) severe diseases in the internal organs, including the heart, liver, and kidney; 6) allergic to the study drug; 7) acupuncture syncope; 8) were taking medications that may interfere in the efficacy assessment of the study therapies, including nimodipine, citicoline sodium capsules, and/or nicergoline tablets at the time of the enrollment interview or within 30 days before the enrollment interview.

### Randomization, allocation, and blinding

Eligible patients were randomized into the following 3 groups at 1:1:1 ratio: nimodipine alone, acupuncture alone, nimodipine and acupuncture. The patient allocation sequence was generated using a complete randomization procedure. The random number and the associated allocated treatment were kept in sequentially numbered sealed opaque envelopes. The person that generated the randomization sequence was not a participating investigator and not involved in patient enrollment and assessment. In this trial, patients were not blinded for the treatment allocation. Physicians that prescribed the study medication and acupuncture therapy were not blinded for the treatment allocation. Both the trained assessor, who evaluated patient MoCA score, and the statistician, who conducted the data analysis, were blinded to the treatment allocation. The treatment allocation was exposed to the participating investigators after the completion of data analysis.

### Interventions

During the study, eligible patients were permitted to continue their existing medications, including drugs to inhibit platelet aggregation, lipid-lowering drugs, anti-hypertensive drugs, and hypoglycemic agents. Patients were required to document the medication use in details. Eligible patients were treated with one of the following 3 therapies: nimodipine tablet (Bayer healthcare company, H20003010, 30 mg per tablet) alone, acupuncture alone, and nimodipine + acupuncture. The dosage of the nimodipine therapy was one tablet per time and 3 times daily for 3 months. The dosage of acupuncture therapy was one 30-min acupuncture section daily 6 days per week from Monday to Saturday for 3 months [17–19]. Disposable sterile acupuncture needles (Brand name: Hua Tuo, size: 0.25 × 40 mm, Suzhou Medical instruments Co., Ltd, China) were used. A total of 12 acupoints were treated in the acupuncture therapy, including Baihui (DU20), Sishencong (EX-HN1), Sibai (ST2), Fengchi (GB20), Wangu (GB12), Tianzhu (BL10), Renzhong (DU26), Shenmen (HT7), Neiguan (PC6), Fenglong (ST40), Sanyinjiao (SP6), and Taichong (LR3) [20, 21]. The skin areas of the acupoints were sterilized by a wipe with 75 % ethanol.

Distinct acupuncture needling technique was applied on different acupoints. Horizontal insertion with one Chinese cun (approximately 3.3 cm) in depth was applied on Baihui (DU20) and Sishencong (EX-HN1). Oblique insertion was used for the acupoint Sibai (ST2) and the needled was inserted 0.5 cun (approximately 1.65 cm) in depth. The needles in the 3 acupoints were twirled gently for 30 s. At the acupoints Fengchi (GB20), Wangu (GB12), and Tianzhu (BL10), vertical insertion with a 1 to 1.5 cun (3.3 to 4.95 cm) in depth was used and the needles were also twirled gently for 30 s. A needle was inserted obliquely with 0.3 to 0.5 cun in depth toward the nasal septum at Renzhong (DU26), and heavy bird-pecking needling was applied until tear formation was observed in patients’ eyes. Vertical insertion with a depth of 0.5 to 1 cun was applied at Neiguan (PC6), and the needle was twirled, lifted, and thrust gently for one minute. At Shenmen (HT7) and Sanyinjiao (SP6), needles were inserted vertically with a depth of 0.2–0.5 cun and 0.5–1.0 cun, respectively. The needles were twirled gently 30 s. Needles were inserted vertically with a depth of 1.0 to 1.5 cun at Fenglong (ST40) and Taichong (LR3), and the needles were twirled, lifted, and thrust gently for one minute. Needles were kept in all of the acupoints for 30 min.

### Outcome assessment

Patients’ cognitive function was assessed by the MoCA test, and MoCA score was used to evaluate the efficacy of the therapies. The MoCA test and the instructions in Chinese are available for download from the following official website of the Montreal Cognitive Assessment: http://www.mocatest.org/wp-content/uploads/2015/tests-instructions. The MoCA test is displayed in Additional file 1: Table S1. Trained assessors evaluated patients’ MoCA score at the enrollment interview, at the end of the 3-month therapy, and at the 3-month post-treatment follow-up. The efficacy index was calculated based on the following equation: (post-treatment or follow-up MoCA score – baseline MoCA score) ÷ baseline MoCA score × 100 %. Efficacy index ≥12 % was considered effective response and <12 % was considered ineffective response.

### Adverse events

Adverse events (AEs) that may or may not be associated with the study therapies, including abnormal gastrointestinal reactions, cardiovascular events, allergic reactions, and other medical conditions were recorded.

### Sample size determination

Previous studies have shown that the rate of effective response was approximately 60 % and 80 % in patients with MCI receiving nimodipine alone and acupuncture alone, respectively [17]. We assumed that the rate of effective response to combination of nimodipine and acupuncture would be ≥ 85 %. Under the assumption of a significant level of 0.05 (one-side), 80 % power, and 15 % data loss, a total of 120 patients were required. Thus, 126 patients (42 patients in each group) were enrolled in this study.

### Statistical analysis

Patients that had poor compliance to the study protocol, failed to come back to the follow-up, or developed severe adverse reactions were removed from the analysis. Per-protocol (PP) dataset was used for statistical analysis. Continuous variables are presented as mean ± standard deviation (SD). Count variables are presented as number of cases or percentage. One-way ANOVA was used to compare baseline BMI, and Kruskal-Wallis rank sum test was used to compare age, sex distribution, and MoCA baseline score. For intra-group comparison of MoCA score improvement at the end of 3-month therapy versus that at the follow-up, Kruskal-Wallis rank sum test was used for the acupuncture alone group; independent sample t-test was used for the nimodipine alone and nimodipine + acupuncture groups. Kruskal-Wallis rank sum test was used for comparison of percentage improvement of MoCA score at the end of 3-month therapy versus that at the follow-up in each group. Kruskal-Wallis rank sum test was used for inter-group comparison of MoCA score improvement; Nemenyi test was used for 2-group comparison. Pearson chi-square test was used to compare the proportion of effective response of the 3 groups. The statistical analysis software SPSS 18.0 was used. *P* value was 2-sided and *P* < 0.05 was considered significantly different.

## Results

### Baseline data

**Fig. 1:**
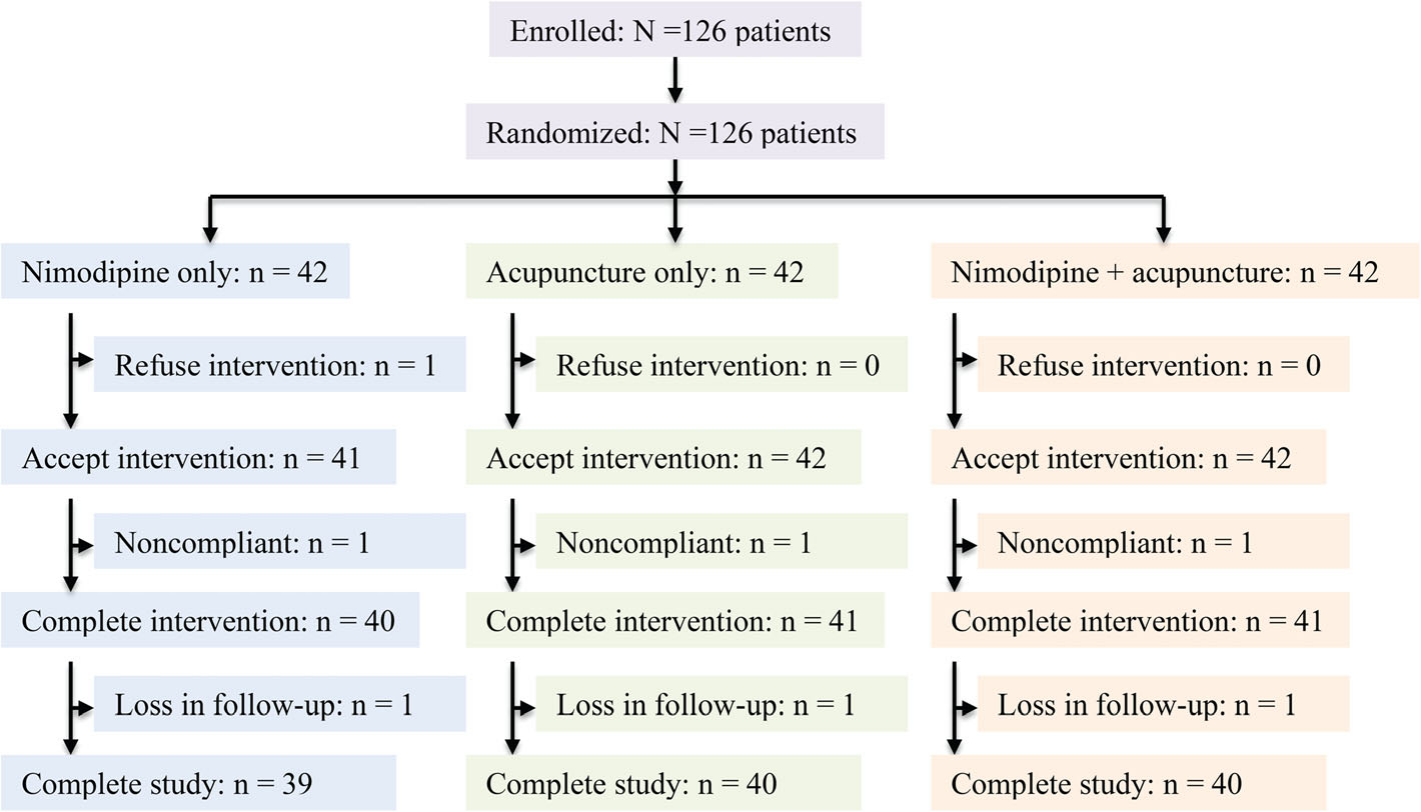
CONSORT patient flow diagram

A total of 126 patients were recruited from April 2013 to June 2014 and randomized into the 3 groups (42 patients per group). After the 3-month post-treatment follow-up, data of 39, 40, and 40 patients from nimodipine alone, acupuncture alone, and nimodipine + acupuncture group, respectively were analyzed. Thus, the PP dataset included 119 patients, and the dropout rate was 5.6 % (7/126). The CONSORT flow diagram is displayed in Fig. 1b. Baseline data showed that sex distribution and body mass index were comparable in the 3 groups (Table 1). However, age was significantly different among the 3 groups (*P* = 0.021), and patients in the nimodipine alone group appeared to have younger mean age than patients in acupuncture alone and acupuncture + nimodipine group (Table 1). The baseline MoCA score was not significantly different among the 3 groups (*P* = 0.242, Table 1).

**Table 1:** Baseline data

	Nimodipine alone, *n* = 39	Acupuncture alone, *n* = 40	Nimodipine + acupuncture, *n* = 40	*P* value
Age (years), mean (SD)	60.6 (6.7)	64.4 (7.7)	65.2 (7.1)	0.021
BMI, mean (SD)	23.2 (2.2)	24.2 (2.2)	23.9 (2.9)	0.220
Men, *n* (%)	26 (66.7)	30 (75.0)	26 (65.0)	0.584
HDL (mmol/L), mean (SD)	1.1 (0.2)	1.1 (0.3)	1.1 (0.2)	
LDL (mmol/L), mean (SD)	2.5 (1.0)	2.6 (0.7)	2.6 (0.8)	
Triglycerides (mmol/L), mean (SD)	1.6 (0.7)	1.8 (1.4)	1.5 (0.7)	
Fasting blood glucose (mmol/L), mean (SD)	6.2 (2.1)	5.7 (2.0)	5.7 (2.0)	
MoCA				
Mean (SD)	21.1 (4.3)	21.8 (3.5)	20.5 (3.9)	0.242
Median	22	22	22	
Min, Max	7, 26	10, 25	11, 25	

*SD* standard deviation, *BMI* body mass index, *HDL* high-density lipoprotein, *LDL*, low-density lipoprotein

### Efficacy assessment

In each group, the mean MoCA score increased significantly at the end of 3-month treatment compared with the baseline values (All *P* < 0.05, Table 2), and further increased and remained at a significantly higher level at the 3-month follow-up (All *P* < 0.05, Table 2). The mean percentage MoCA improvement at the follow-up was higher than that at the end of 3-month treatment in each group (Table 2). Particularly, in the combination therapy group, the percentage MoCA score improvement at follow-up was significantly higher than that at the end of the combination therapy (*P* = 0.037, Table 2). These data suggest that all 3 therapies may improve cognitive function and the improvement appear to be enhanced and sustained at least for 3 months after the therapies.

**Table 2:** Intra-group comparison of the effect of the therapies on MoCA score

	Nimodipine alone			Acupuncture alone			Nimodipine + Acupuncture		
	*n* = 39, Mean (SD)			*n* = 40, Mean (SD)			*n* = 40, Mean (SD)		
	MoCA	Increase	%	MoCA	Increase	%	MoCA	Increase	%
Baseline	21.1 (4.3)	n/a	n/a	21.8 (3.5)	n/a	n/a	20.5 (3.9)	n/a	n/a
After treatment	23.5 (4.6)^a^	2.4 (2.1)	12.7 (14.1)	25.4 (4.1)^a^	3.6 (2.4)	16.8 (13.1)	24.5 (3.3)^a^	4.0 (2.0)^b^	21.6 (14.3)^c^
At follow-up	24.2 (4.6)^a^	3.1 (1.8)	15.8 (10.9)	26.1 (3.6)^a^	4.3 (2.3)	20.9 (13.8)	26.0 (2.8)^a^	5.5 (2.2)^b^	30.2 (19.7)^c^

MoCA score increase was calculated as: MoCA score at the end of the therapy or at the follow-up – baseline MoCA score. MoCA % increase was calculated as: MoCA score increase ÷ baseline MoCA score × 100 %. ^a^represents significant different between the indicated score vs. the baseline MoCA score in each group. ^b^represents significant different between the indicated values (*P* = 0.002). ^c^represents significantly different between the indicated values (*P* = 0.037)

**Fig. 2:**
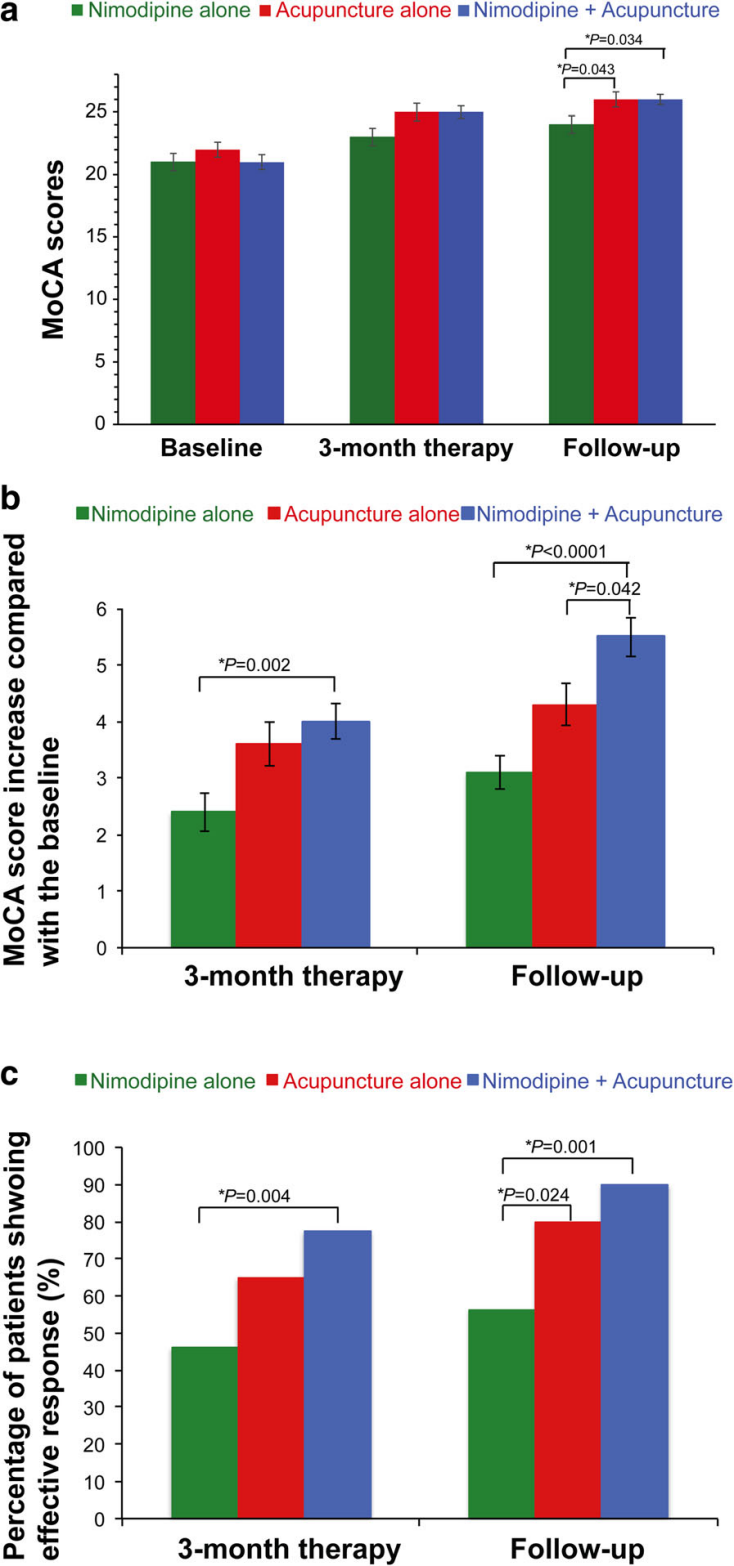
Comparison of MoCA score improvement and proportion of effective response of the 3 groups. **a** MoCA scores of the three patient groups. **b** Comparison of MoCA score improvement of the 3 groups. Kruskal-Wallis rank sum test was used to compare the 3 groups; Nemenyi test was used for 2-group comparison. **c** Comparison of the proportion of effective response of the 3 groups. Pearson chi-square test was used. *represent significant difference, and *P* < 0.05 was considered statistically significantly different

The actual MoCA scores at follow-up of the acupuncture alone group (26.1 ± 3.6, *P* = 0.043) and nimodipine + acupuncture groups (26.0 ± 2.8, *P* = 0.034) were significantly higher than that of the nimodipine alone group (24.2 ± 4.6, Fig. 2a). However, the MoCA scores at the end of 3-month therapy were not significantly different in the three patient groups (Fig. 2a). Inter-group comparison revealed that the mean MoCA score improvement after 3-month acupuncture alone (3.6 ± 2.4) was higher than that after 3-month nimodipine alone treatment (2.4 ± 2.1) although the difference was not statistically significant (*P* = 0.091, Fig. 2b). In contrast, The MoCA score improvement after 3-month combination therapy (4.0 ± 2.0) was significantly greater than that after nimodipine alone treatment (*P* = 0.002, Fig. 2b). At the post-treatment 3-month follow-up, the MoCA score improvement in acupuncture alone (4.3 ± 2.3) was not significantly different from that (3.1 ± 1.8) in nimodipine alone group (*P* = 0.070, Fig. 2b). However, the MoCA score improvement at the post-treatment 3-month follow-up in the combination therapy group (5.5 ± 2.2) was significantly higher than both acupuncture alone (*P* = 0.042) and nimodipine alone (*P* < 0.0001, Fig. 2b) groups. Thus, the combination therapy of nimodipine and acupuncture appears to have superior efficacy on post-cerebral infarction MCI to nimodipine monotherapy and acupuncture monotherapy.

In addition, at the end of 3-month treatment, significantly higher proportion of patients in the combination therapy group (77.5 %) showed effective response (≥12 % increase in MoCA score compared with the baseline MoCA) than the nimodipine alone group (46.2 %, *P* = 0.004, Fig. 2c). At the post-treatment 3-month follow-up, the proportion of patients that showed effective response increased to 80 % and 90 % for the acupuncture alone group and the combination therapy group, respectively, and these values were significantly higher than that in the nimodipine alone group (56.4 %, all *P* < 0.05, Fig. 2c). No AE was reported during the study. The original data are presented in the Additional file 2: Table S2.

## Discussion

In this randomized controlled clinical trial, 3-month nimodipine monotherapy, acupuncture monotherapy, and combination therapy of nimodipine plus acupuncture improved cognitive function significantly in patients with post-cerebral infarction MCI, and the cognitive improvement was sustained and even became more apparent 3 months after the treatments. Notable, compared with the monotherapies, the combination therapy was associated the best cognitive improvement, suggesting that nimodipine and acupuncture may result in additive neurological benefit in patients with post-cerebral infarction MCI.

These findings are consistent with the results from previous trials assessing the therapeutic value of acupuncture in cognitive impairment. Our previous clinical trial showed that MoCA score improvement after 3-month combination therapy of nimodipine plus acupuncture was 1.88 folds of that after nimodipine monotherapy group [22]. Similarly, this current study found that the MoCA score improvement in the combination therapy group was 1.66 folds of that in the nimodipine monotherapy group at the end of the treatment and further increased to 1.77 folds at post-treatment 3-month follow-up. Different types of acupuncture, such as electroacupuncture and body acupuncture, different types of conventional therapy, including nimodipine, donepezil, and cognitive function training, and various efficacy evaluation system, such as MoCA, Mini-Mental State Examination (MMSE), Modified Barthel Index, and Wechsler Memory Scale have been used in clinical trials to compare the efficacy of combination of acupuncture plus conventional therapy versus conventional therapy alone in patients with vascular MCI. Cao et al. conducted a meta-analysis on 12 randomized controlled trials and found that combination therapy consistently showed superior efficacy on cognitive improvement to conventional therapies regardless of the types of acupuncture therapy, conventional therapy, and efficacy evaluation system that were used in the trials although most of the included studies suffered poor quality in methodology and high risk of bias [14]. This conclusion is further supported by a more recent meta-analysis that compared the efficacy of combination of acupuncture plus conventional therapy versus conventional therapy alone in patients with post-stroke cognitive impairment [23].

In this current study, the improvement in MoCA score after acupuncture monotherapy was higher than that after nimodipine monotherapy although the difference was not statistically significant. However, significantly higher proportion of patients achieved ≥12 % increase in MoCA score after acupuncture monotherapy than after nimodipine monotherapy. These findings suggest that acupuncture may be more effective than nimodipine in patients with post-cerebral infarction MCI. Similarly, previous studies have demonstrated that both electroacupuncture and body acupuncture monotherapy improved MMSE score or MoCA score at significantly higher extent than nimodipine alone [14, 24].

The molecular and cellular mechanism underlying the beneficial effect of acupuncture on neurological outcome has been investigated in animal models. Lee et al. used scopolamine to induce cognitive impairment in rats and found that acupuncture stimulation at Baihui (DU20) acupoint improved rat memory significantly and restored the mRNA expression of molecules that are involved in memory function in hippocampus such as cAMP-response element-binding protein (CREB) [25]. Similarly, Li et al. also found that acupuncture increased the expression of CREB and phosphorylated CREB and improved memory function in rats with vascular dementia induced by homologous blood clot emboli [26]. Furthermore, acupuncture has been shown to significantly increase the number of neurons in the hippocampal CA1 area of rats with vascular dementia [27, 28]. Magnetic resonance imaging (MRI) revealed that acupuncture activated neurons in cognitive-related cortical area in patients with MCI [29–31]. These preclinical and clinical studies indicate that acupuncture may improve cognitive function by activating neurons, reversing brain damage-associated alteration of molecules, such as the CREB pathway, and increasing the number of neurons in hippocampus.

There are several limitations in this study. Only MoCA scale was used to assess the cognitive function in this current study. Although compared with comprehensive neuropsychological battery, MoCA is short and has good sensitive and specificity to MCI, additional evaluation test such as Addenbrooke’s Cognitive Examination–Revised would improve the evaluation accuracy [15]. The duration of acupuncture therapy and follow-up time in this current study was only 3 months, respectively. Long-term effect of acupuncture on vascular MCI remains unknown. Moreover, patients in the acupuncture alone and the combination therapy groups were significantly older than patients in the nimodipine alone group. This non-uniform age distribution may be associated with the small sample size and single center nature of this current study. Thus, multi-center large-scale studies with better randomization scheme and comprehensive efficacy evaluation method are required to further verify the conclusion. Furthermore, the improvements across the therapies in the current study could be partially attributable to a spontaneous recovery. A placebo control group, in which patients received no treatments, would be necessary to investigate the spontaneous recovery. However, because the participating patients were likely in a critical therapeutic window (0.5 to 6 months post-infarction) for effective interventions to attenuate the MCI and the potential risk of a deterioration of the MCI if left untreated may be high, inclusion of a placebo control group in the current prospective randomized controlled trial may highly likely be disapproved by the Institutional Review Board. In addition, even if a spontaneous recovery existed, it would theoretically affect the three patient group equally because of the randomized design in the current study. Thus, we did not include such placebo control. Certainly, the current study cannot exclude the possibility that some patients with post-cerebral infarction MCI may recover spontaneously. Spontaneous recovery from post-stroke cognitive impairment has been reported previously [32–37]. Rasquin et al. followed up 118 patients with a first ever cerebral stroke for 2 years, and they found that 20.3 % of the patients had reversible MCI and most of the recovery occurred between one-month and 6-month post-stroke [37]. They also discovered that higher baseline MMSE scores, which was determined at post-stroke one month, and female sex were independent predictors of the recovery [37].

## Conclusions

The findings of this current study suggest that acupuncture may be used as an additional therapy to conventional pharmacological treatment to further improve the clinical outcomes of patients with post-cerebral infarction MCI. Long-term and large scaled studies with more comprehensive efficacy evaluation system are still required to further verify the conclusion.

## Additional files

Additional file 1: MoCA test in Chinese. (PDF 947 kb) Additional file 2: Patients' MoCA score. (XLSX 90 kb)

## Abbreviations

AE: Adverse event
MCI: Mild cognitive impairment
MMSE: Mini-mental state examination
MoCA: Montreal cognitive assessment
PP: Per-protocol
VCI: Vascular cognitive impairment

## References

[1] Sun JH, Tan L, Yu JT (2014). Post-stroke cognitive impairment: epidemiology, mechanisms and management. Ann Transl Med.

[2] Makin SD, Turpin S, Dennis MS, Wardlaw JM (2013). Cognitive impairment after lacunar stroke: systematic review and meta-analysis of incidence, prevalence and comparison with other stroke subtypes. J Neurol Neurosurg Psychiatry.

[3] Wilkinson D, Doody R, Helme R, Taubman K, Mintzer J, Kertesz A (2003). Donepezil in vascular dementia: a randomized, placebo-controlled study. Neurology.

[4] Black S, Román GC, Geldmacher DS, Salloway S, Hecker J, Burns A (2003). Efficacy and tolerability of donepezil in vascular dementia: positive results of a 24-week, multicenter, international, randomized, placebo-controlled clinical trial. Stroke.

[5] Román GC, Salloway S, Black SE, Royall DR, Decarli C, Weiner MW (2010). Randomized, placebo-controlled, clinical trial of donepezil in vascular dementia: differential effects by hippocampal size. Stroke.

[6] Rockwood K, Mitnitski A, Black SE, Richard M, Defoy I, VASPECT study investigators (2013). Cognitive change in donepezil treated patients with vascular or mixed dementia. Can J Neurol Sci.

[7] Auchus AP, Brashear HR, Salloway S, Korczyn AD, De Deyn PP, Gassmann-Mayer C (2007). Galantamine treatment of vascular dementia: a randomized trial. Neurology.

[8] Ballard C, Sauter M, Scheltens P, He Y, Barkhof F, van Straaten EC (2008). Efficacy, safety and tolerability of rivastigmine capsules in patients with probable vascular dementia: the VantagE study. Curr Med Res Opin.

[9] Wilcock GK (2003). Memantine for the treatment of dementia. Lancet Neurol.

[10] Gorelick PB, Scuteri A, Black SE, Decarli C, Greenberg SM, Iadecola C (2011). Vascular contributions to cognitive impairment and dementia: a statement for healthcare professionals from the American Heart Association/American Stroke Association. Stroke.

[11] Allen GS, Ahn HS, Preziosi TJ, Battye R, Boone SC, Boone SC (1983). Cerebral arterial spasm--a controlled trial of nimodipine in patients with subarachnoid hemorrhage. N Engl J Med.

[12] Tomassoni D, Lanari A, Silvestrelli G, Traini E, Amenta F (2008). Nimodipine and its use in cerebrovascular disease: evidence from recent preclinical and controlled clinical studies. Clin Exp Hypertens.

[13] López-Arrieta JM, Birks J (2002). Nimodipine for primary degenerative, mixed and vascular dementia. Cochrane Database Syst Rev.

[14] Cao H, Wang Y, Chang D, Zhou L, Liu J (2013). Acupuncture for vascular mild cognitive impairment: a systematic review of randomised controlled trials. Acupunct Med.

[15] Pendlebury ST, Mariz J, Bull L, Mehta Z, Rothwell PM (2012). MoCA, ACE-R, and MMSE versus the National Institute of Neurological Disorders and Stroke-Canadian Stroke Network Vascular Cognitive Impairment Harmonization Standards Neuropsychological Battery after TIA and stroke. Stroke.

[16] Gauthier S, Reisberg B, Zaudig M, Petersen RC, Ritchie K, Broich K (2006). Mild cognitive impairment. Lancet.

[17] Lu X, Liang Y, Tang D, Lv P, Cai D (2014). A review of the correlation between the time for keeping needles and acupuncture efficacy. Asia-Pacific Traditional Med.

[18] Zhang T, Guan F, Le C, Hao C, Liu X, Ji L (2009). The correlation between the time for keeping needles and the efficacy of using acupuncture to treat stroke. Acupunct Res.

[19] Li F, Cai RL, Zhai L, Gao K, Yang J (2013). Impacts of acupuncture at Jing-well points on the differentiated meridians and temple-three-needle therapy on P300 of patients with early vascular cognitive impairment. Zhongguo Zhen Jiu.

[20] Xia Y, Fan X (2013). Expert’s opinion on prevention and treatment of vascular cognitive impairment: An interview with Dr. Xuemin Shi. J Clin Acupunct Moxibustion.

[21] Wang C (2007). The efficacy of acupuncture to treat vascular cognitive impairment. J Emerg Traditional Chinese Med.

[22] Yang H, Zhang B, Liu T, Zheng J (2015). Efficacy of acupuncture in combination with medicine for mild cognitive impairment after cerebral infarction: a randomized controlled trial. World J Acupuncture-Moxibustion.

[23] Liu F, Li ZM, Jiang YJ, Chen LD (2014). A meta-analysis of acupuncture use in the treatment of cognitive impairment after stroke. J Altern Complement Med.

[24] Zhang H, Zhao L, Yang S, Chen Z, Li Y, Peng X (2013). Clinical observation on effect of scalp electroacupuncture for mild cognitive impairment. J Tradit Chin Med.

[25] Lee B, Sur B, Shim J, Hahm DH, Lee H (2014). Acupuncture stimulation improves scopolamine-induced cognitive impairment via activation of cholinergic system and regulation of BDNF and CREB expressions in rats. BMC Complement Altern Med.

[26] Li QQ, Shi GX, Yang JW, Li ZX, Zhang ZH, He T (2015). Hippocampal cAMP/PKA/CREB is required for neuroprotective effect of acupuncture. Physiol Behav.

[27] Li F, Yan CQ, Lin LT, Li H, Zeng XH, Liu Y (2015). Acupuncture attenuates cognitive deficits and increases pyramidal neuron number in hippocampal CA1 area of vascular dementia rats. BMC Complement Altern Med.

[28] Wang XR, Shi GX, Yang JW, Yan CQ, Lin LT, Du SQ (2015). Acupuncture ameliorates cognitive impairment and hippocampus neuronal loss in experimental vascular dementia through Nrf2-mediated antioxidant response. Free Radic Biol Med.

[29] Chen S, Xu M, Li H, Liang J, Yin L, Liu X (2014). Acupuncture at the Taixi (KI3) acupoint activates cerebral neurons in elderly patients with mild cognitive impairment. Neural Regen Res.

[30] Wang Z, Nie B, Li D, Zhao Z, Han Y, Song H (2012). Effect of acupuncture in mild cognitive impairment and Alzheimer disease: a functional MRI study. PLoS One.

[31] Feng Y, Bai L, Ren Y, Chen S, Wang H, Zhang W (2012). FMRI connectivity analysis of acupuncture effects on the whole brain network in mild cognitive impairment patients. Magn Reson Imaging.

[32] Tham W, Auchus AP, Thong M, Goh ML, Chang HM, Wong MC (2002). Progression of cognitive impairment after stroke. One year results from a longitudinal study of Singaporean stroke patients. J Neurol Sci.

[33] Ballard C, Rowan E, Stephens S, Kalaria R, Kenny RA (2003). Prospective follow-up study between 3 and 15 months after stroke: improvements and decline in cognitive function among dementia-free stroke survivors N75 years of age. Stroke.

[34] Hochstenbach JB, den Otter R, Mulder TW (2003). Cognitive recovery after stroke: a 2-year follow-up. Arch Phys Med Rehabil.

[35] Patel M, Coshall C, Rudd AG, Wolfe CD (2003). Natural history of cognitive impairment after stroke and factors associated with its recovery. Clin Rehabil.

[36] Desmond DW, Moroney JT, Sano M, Stern Y (1996). Recovery of cognitive function after stroke. Stroke.

[37] Rasquin SM, Lodder J, Verhey FR (2005). Predictors of reversible mild cognitive impairment after stroke: a 2-year follow-up study. J Neurol Sci.

