# Supplementary material for: Efficacy and safety assessment of acupuncture and nimodipine to treat mild cognitive impairment after cerebral infarction: a randomized controlled trial
Source: BMC Complement Altern Med. 2016 Sep 13;16(1):361. doi: 10.1186/s12906-016-1337-0 (PMC5022140; doi:10.1186/s12906-016-1337-0)
Supplement: Additional file 1: — MoCA test in Chinese. (PDF 947 kb) [file 12906_2016_1337_MOESM1_ESM.pdf]

Montreal Cognitive Assessment (MoCA) Beijing Version

蒙特利尔认知评估北京版

出生日期：
教育水平：
性　　别：
姓名：
检查日期：

视空间与执行功能

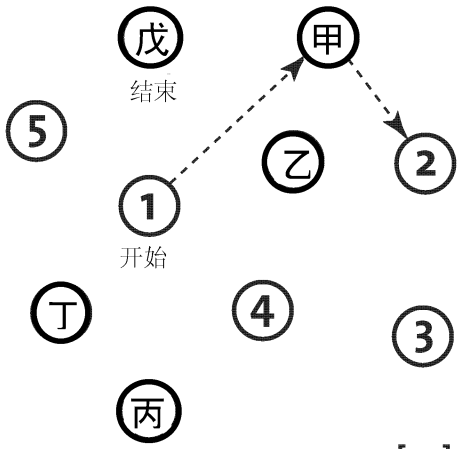

[ ]

复制立方体

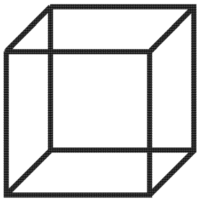

[ ]

画钟表（11点过10分）（3分）

[ ] 轮廓　[ ] 数字　[ ] 指针

得分 ___/5

命名

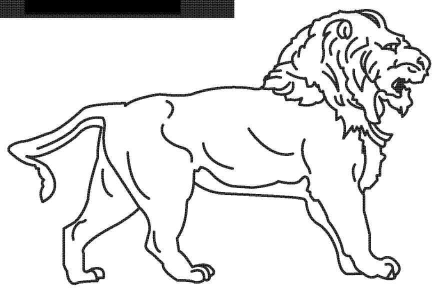

[ ]

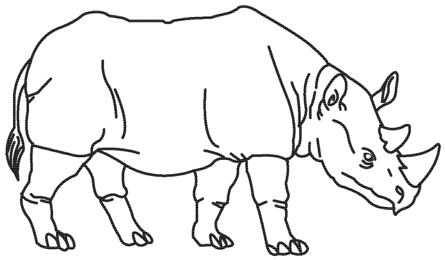

[ ]

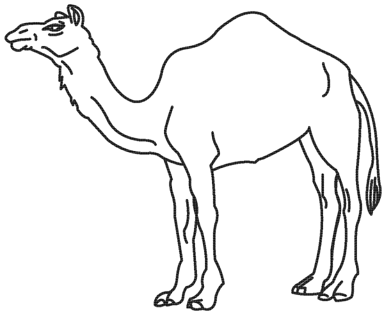

[ ]

___/3

记忆

读出下列词语,而后由患者重复
上述过程重复2次
5分钟后回忆

	面孔	天鹅绒	教堂	菊花	红色
第一次					
第二次					

不计分

注意

读出下列数字,请患者重复（每秒1个）

顺背 [ ] 2 1 8 5 4
倒背 [ ] 7 4 2

___/2

读出下列数字,每当数字1出现时,患者必须用手敲打 一下桌面,错误数大于或等于2个不给分

[ ] 5 2 1 3 9 4 1 1 8 0 6 2 1 5 1 9 4 5 1 1 1 4 1 9 0 5 1 1 2

___/1

100连续减7　[ ] 93　[ ] 86　[ ] 79　[ ] 72　[ ] 65

4-5个正确给3分,2-3个正确给2分,1个正确给1分,全都错误为0分

___/3

语言

重复: 我只知道今天张亮是来帮过忙的人 [ ]
狗在房间的时候，猫总是躲在沙发下面 [ ]

___/2

流畅性: 在1分钟内尽可多的说出动物的名字 [ ] ____ (N ≥ 11 名称)

___/1

抽象

词语相似性:如香蕉-桔子=水果 [ ] 火车-自行车 [ ]手表-尺子

___/2

延迟回忆

回忆时不能提示	面孔	天鹅绒	教堂	菊花	红色	仅根据非提示回忆计分
	[ ]	[ ]	[ ]	[ ]	[ ]	
选项：分类提示						
选项：多选提示						

___/5

定向

[ ] 日期　[ ] 月份　[ ] 年代　[ ] 星期几　[ ] 地点　[ ] 城市

___/6

总分 ___/30

© Z.Nasreddine MD Version November 7, 2004

Beijing version 26 August, 2006 translated by Wei Wang & Hengge Xie
www.mocatest.org
